# Adipose tissue biglycan as a potential anti-inflammatory target of sodium salicylate in mice fed a high fat diet

**DOI:** 10.1186/1476-9255-9-15

**Published:** 2012-04-25

**Authors:** Venkata J Adapala, Meliza Ward, Kolapo M Ajuwon

**Affiliations:** 1 Department of Animal Sciences, Purdue University, West Lafayette, IN, 47907-2054, USA; 2 Interdepartmental Nutrition Program, Purdue University, West Lafayette, IN, 47907-2054, USA

## Abstract

**Background:**

Inflammation in adipose tissue (AT) during obesity causes impaired AT function. Although multiple extracellular matrix (ECM) proteins are expressed in AT their potential role in adipose tissue inflammation is unclear. Biglycan, a pro-inflammatory ECM gene, is highly enriched in adipose tissue. However, whether it is correlated with adipose tissue inflammation is unknown. We provide evidence in support of a strong association between biglycan expression and inflammatory status of adipose tissue.

**Methods:**

C57BL6 mice were fed either a control (10% fat calories) or a high fat diet (HFD) (60% fat calories) for 8 weeks. Adipose tissue was analyzed for the expression of biglycan, IL-6 and TNFα. Biglycan knockout or wild type were also fed a high fat diet for 8 weeks and the expression of inflammatory genes in the mesenteric adipose tissue was examined. To test anti-inflammatory treatment on biglycan expression, a group of mice were fed either the low fat or high fat diet for eight weeks supplemented with either saline or sodium salicylate @ 25mg/100ml in their drinking water.

**Results:**

Mice on HFD had an increase in ECM genes (BGN and COL1A1), inflammatory genes (IL-6 and TNFα) in both the subcutaneous and epididymal depots. However, correlation analysis only shows a positive correlation between biglycan, IL-6 and TNFα expression. In addition, lower expression of IL-6 and CD68 was found in the mesenteric adipose tissue of biglycan knockout mice compared to the wild type. Sodium salicylate treatment reduced subcutaneous adipose tissue expression of BGN, COL1A1, and COL6A1 and a concurrent downregulation of TNFα and IL-6 and TLR4 expression. Salicylate also lowered the serum TGFβ1 levels.

**Conclusion:**

Biglycan expression correlates with adipose tissue inflammation, especially in the subcutaneous depot compared to the epididymal depot. This is supported by the greater effect of sodium salicylate in attenuating both inflammatory and ECM gene expression the subcutaneous adipose depot compared to the epididymal depot. These results show that inflammatory state may explain the induction of biglycan, and perhaps, other ECM genes in adipose tissue.

## Background

Obesity is associated with chronic low-grade inflammation in adipose tissue which predisposes to metabolic disorders like insulin resistance and type II diabetes and cardiovascular complications [1-3]. Although multiple ECM proteins are present in adipose tissue, it is currently unknown if there is a link between these proteins and adipose tissue inflammation. Extracellular matrix proteins, apart from providing mechanical support to adipocytes and other cells, also participate in a variety of signaling events including activation of inflammatory signals [4,5]. This evidence is supported in mouse models of obesity such as the ob/ob and the db/db mice which have elevated expression of inflammatory markers and ECM genes [6]. Furthermore, phenotypic changes induced by the action of proinflammatory mediators on preadipocytes lead to increased synthesis of ECM components in the subcutaneous adipose tissue of obese human subjects [7]. Information is currently lacking on the role of specific ECM proteins in the regulation of AT inflammation. Biglycan is an ECM protein with proinflammatory effect [8]. Biglycan activates inflammatory pathways by signaling through toll-like receptor (TLR) 2 and 4 in macrophages leading to secretion of TNFα and mature IL-1β [8]. A more recent discovery indicates that biglycan activates the inflammasome pathway by inducing an interaction between the TLR2/4 and the purinergic P2X(4)/P2X(7) receptors, which are part of the nucleotide-binding oligomerization domain-like receptors (NLRP3/ASC) [5]. This interaction is considered necessary for biglycan induction of IL-1β in macrophages. As shown with receptor knockout mice, biglycan is able to activate these inflammatory pathways through direct binding to these receptors, thus triggering downstream signaling pathways [5,8]

We have recently shown that biglycan is highly expressed in subcutaneous adipose tissue of obese human subjects that also display elevated expression of inflammatory cytokine TNFα and absence of biglycan is associated with reduced expression of TNFα in mice [9]. Although the exact stimulus for the induction of biglycan expression in obesity is unknown, because biglycan expression can be induced by inflammatory cytokines [8], saturated fatty acids, which are also proinflammatory, or inflammatory cytokines may mediate the increased expression of biglycan in AT in obesity. In this report, we have employed an anti-inflammatory treatment with sodium salicylate to determine if therapeutic reduction of AT inflammation is accompanied by decreased ECM expression.

The therapeutic effects of sodium salicylate are well known from the late 19^th^ century. However, limited studies have investigated the detailed molecular targets of salicylate, especially in AT. The anti-inflammatory effect of salicylate compounds is partly through the inhibition of cyclooxygenase (COX) enzymes involved in prostaglandin synthesis [10]. Later studies also showed that this anti-inflammatory action included activation of p38 MAPK [11] and inhibition of nuclear transcription factor kappa B (NF-kB) and its upstream activators [12,13]. High doses of salicylate compounds inhibit the translocation of transcription factor NF-kB into the nucleus by preventing the degradation of IkBα [12] through the specific inhibition of IKKβ kinase activity [14]. Salicylates in high doses can also exert metabolic benefits such as improvement in fasting glucose and insulin levels in both obese and diabetic subjects [15-17] and lowering of blood triglyceride and free fatty acid concentrations [15,18] and increase in insulin sensitivity [10]. We show herein that reduction in AT inflammation corresponds to reduced biglycan expression. Thus, inflammatory state is a key factor responsible for AT ECM expression. Understanding the connection between biglycan and adipose tissue inflammation will expand our understanding of the role of ECM components in inflammation.

## Methods

### Animals and experimental design

All mice used for the experiment were housed in the pathogen free facility at the Purdue Small Animal Housing facility. In the first experiment conducted to determine the effect of high fat diet on biglycan and inflammatory gene expression, male C57BL/6J mice (Jackson Laboratories, Bar Harbor, Maine) at 8 weeks of age were divided equally (8 mice per group) between a control diet with 10 kcal % fat (D12450Bi) and a high fat diet with 60 kcal % fat (D12492i) (Research Diets, New Brunswick, NJ) and fed for 8 weeks. Thereafter, epididymal AT was analyzed for biglycan and inflammatory gene expression. The second experiment was carried out to test the anti-inflammatory effect of salicylate. Thirty-two male C57BL/6J mice at 8 weeks old were assigned to either a control diet (n = 16) or a high fat diet (n = 16) as described above. Each diet group was split into two groups (8 mice per group) and given either plain water or water with sodium salicylate at a dose of 25mg/100 ml. After 8 weeks on treatments, mice were killed with CO_2_ asphyxiation and adipose tissues were snap-frozen in liquid nitrogen and stored at -80°C for gene expression and immunoblotting assays. Additional analysis of effect of biglycan absence on inflammatory gene expression was conducted in AT from biglycan knockout and wild type mice as described previously [9]. All animal protocols were approved by the Purdue Animal Care and Use Committee.

### Glucose tolerance test (GTT)

Animals were fasted 12 hours prior to GTT. Mice were administered a 50% dextrose solution via intraperitoneal (IP) injection at 2.0 g/kg body weight. Tail blood was collected at 0 (basal), 15, 30, 45, 60, and 120 minutes respectively after the glucose injection for the determination of blood glucose concentration with an automatic glucometer (Freestyle, Alameda, CA).

### RNA extraction and quantitative RT-PCR

Total RNA from epididymal and subcutaneous AT and gastrocnemius muscle was extracted using TRIzol® (Invitrogen, Carlsbad, CA) and reverse transcribed with the MMLV reverse transcriptase (Promega, Madison, WI). Real-time PCR was performed using the MyiQ real-time PCR detection system (Bio-Rad, Hercules, CA) with the Faststart SYBR mix (Roche, Indianapolis, IN). Primers were designed from gene sequences in the NCBI database with the Invitrogen primer design software. Gene specific mRNA abundance was determined from the threshold cycle (*C*t) for respective genes and normalized against the *C*t for 18S using the ΔΔCt method. Data are expressed as fold changes relative to a control sample which was also used for correcting run-to-run variations. List of primers is as described previously [9].

### ELISA analysis

Serum was separated from whole blood by centrifugation at 5,000g for 5 minutes. TGFβ1 protein level was measured in the serum samples using Human/Mouse TGFβ1 ELISA kit (eBioscience, San Diego, CA) according to the manufacturer’s instructions.

### Statistical analysis

Data were examined for normality and analyzed using the GLM procedures (SAS Inst. Inc., Cary, NC). Turkey analysis was conducted for mean separation. Differences were considered significant at *P* < 0.05. P values between 0.05 and 0.1 were considered showing a strong tendency for significance. Values in the text are means ± SEM.

## Results

### High fat diet induces biglycan and inflammatory gene expression

**Figure 1:**
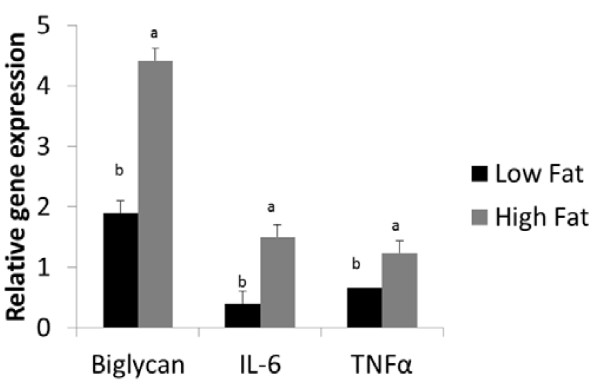
**Effect of diet on the expression of biglycan and inflammatory genes**. Male C57BL/6J mice fed either a low fat diet (10% fat calories) or a high fat diet (HFD) (60% fat calories) for 8 weeks. Epididymal fat RNA was obtained and subjected to RT-PCR analysis. Bars represent means and SE of treatments (n = 8). Bars with different superscripts are different (P < 0.05).

First, we determined the expression of biglycan and inflammatory genes IL-6 and TNFα in epididymal tissue after a high fat diet. As shown in Figure 1, increased expression of biglycan resulted from high fat diet feeding. High fat diet also significantly induced the expression of IL-6 and TNFα. There was also an increase in the expression of other ECM genes such as COL1A1 and COL6A1 (data not shown). To determine if there was any correlation between the expression of biglycan and inflammatory genes, correlation analysis was run between the ECM genes (biglycan, COL1A1 and COL6A1) and the inflammatory genes. Significant correlation was found only between biglycan, IL-6 and TNFα (Table 1). Therefore, we focused on biglycan for the rest of the study.

**Table 1:** Correlation analysis between biglycan expression and selected inflammatory genes in epididymal adipose tissue

Gene	Gene		
	BGN	IL-6	TNFα
BGN		0.38* ^(0.002)^	0.55* ^(0.0001)^
IL-6	0.38*^(0.002)^		0.66*^(0.0001)^
TNFα	0.55*^(0.0001)^	0.66*^(0.0001)^	

*Significant positive correlation between BGN and IL6 (P < 0.002) and TNFα (P < 0.0001).

### Reduced inflammation in biglycan knockout mice

**Figure 2:**
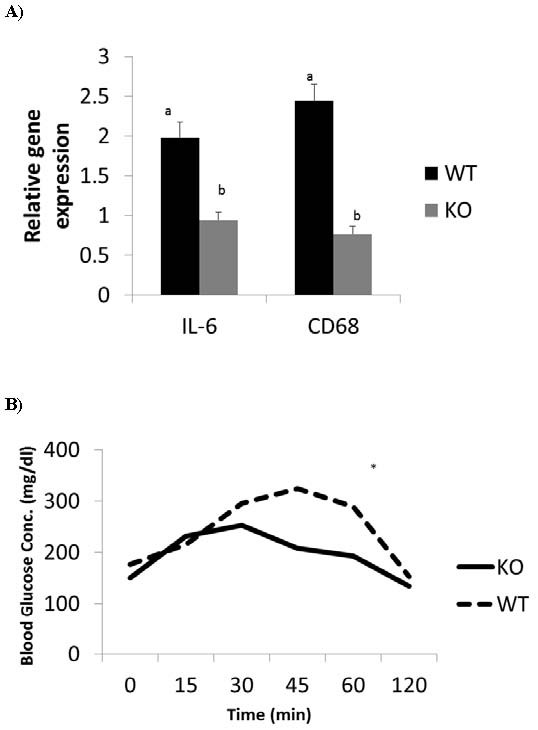
**a. Expression of inflammatory genes in wild type or biglycan knockout mice**. Mice were given a high fat diet for 9 weeks. Expression of inflammatory genes was determined by RT-PCR. Bars represent means (n = 4) and SE of treatments. Bars with different superscripts are different (P < 0.05). **b.** Glucose Tolerance Test (GTT) in wild type (WT) and biglycan null (KO) mice. Mice received intraperitoneal injection of 50% dextrose solution at 2g/kg BW. Blood glucose concentration was measured at 0, 15, 30, 45, 60 and 120 minutes. KO mice are more sensitive than WT mice.*; P < 0.05.

We had earlier reported a decreased expression of TNFα in the adipose tissue of biglycan knockout mice [9]. Therefore we analyzed other inflammatory marker genes, IL-6 and CD68. Similar to TNFα, decreased expression of IL-6 and CD68 was found in biglycan knockout mice compared to the wild type (Figure 2). In addition, glucose tolerance test conducted on the WT and biglycan knockout mice (Figure 2b) revealed better clearance of injected glucose by the knockout mice, an indication of improved metabolic profile in the knockout animals relative to WT. Thus, the absence of biglycan lowers expression of inflammatory makers and leads to improved metabolic profile. This suggests that biglycan could be involved in the development of AT inflammation and metabolic dysregulation in obesity.

### Diet and salicylate effect on growth and body composition

The association between biglycan and inflammatory gene expression suggests that biglycan may be involved in the induction of inflammatory gene expression or vice versa. Indeed, the lower expression of inflammatory genes in the biglycan knockout mice strongly supports a major role for biglycan or biglycan-associated processes in the regulation of inflammatory gene expression. However, to determine whether inflammation is involved in the regulation of biglycan expression, we tested the effect of salicylate, an anti-inflammatory pharmacological compound in mice fed either a control or a high fat diet. As expected, mice on the high fat diet had a higher body weight than those on the control diet (P < 0.0001) (Table 2). However, sodium salicylate had no effect on the body weight of the mice. As predicted, subcutaneous and visceral AT mass of the mice on the HFD was greater than in mice on control diet (P < 0.0001). Although there was no salicylate effect on the overall body weight, mice on the HFD receiving sodium salicylate had a lower subcutaneous AT weight (P < 0.07). This may suggest that sodium salicylate prevents fat deposition in this depot.

**Table 2:** Body composition in mice on control or high fat diet (HFD)

Parameters	Diet				P-Value		
	Control		High Fat					
	Water(n = 8)	Sal.C^10^(n = 8)	Water(n = 8)	Sal.C^5^(n = 8)	Diet	Sal.C	Diet*Sal.C	
Body Weight	33.45 ± 1.18^b^	31.03 ± 1.26^b^	39.33 ± 1.26^a^	40.20 ± 1.26^a^	0.0001	0.51	0.20
Depots							
SubCut^1^(%)	1.60 ± 0.19^c^	1.84 ± 0.20^c^	4.30 ± 0.20^a^	3.27 ± 0.20^b^	0.0001	0.07	0.004
EPI^2^(%)	2.40 ± 0.25^b^	2.94 ± 0.27^b^	5.02 ± 0.27^a^	4.88 ± 0.27^a^	0.0001	0.43	0.20
Retro^3^(%)	0.78 ± 0.10^b^	0.83 ± 0.10^b^	1.75 ± 0.10^a^	1.74 ± 0.10^a^	0.0001	0.87	0.79
Liver(%)	4.09 ± 0.22^a^	4.04 ± 0.23^ab^	3.39 ± 0.23^b^	3.66 ± 0.23^ab^	0.03	0.65	0.50
Muscle^4^(%)	1.31 ± 0.12^a^	1.02 ± 0.12^ab^	1.13 ± 0.12^ab^	0.87 ± 0.15^b^	0.20	0.04	0.91

a,b,c; superscripts represent significant differences in mean values; P < 0.05.

Results are mean ± SEM.

^1^Subcutaneous.

^2^Epididymal.

^3^Retroperitoneal.

^4^Gastrocnemius muscle.

^5^Sodium salicylate.

### Effect of diet and salicylate on the expression of ECM and cytokine genes

**Figure 3:**
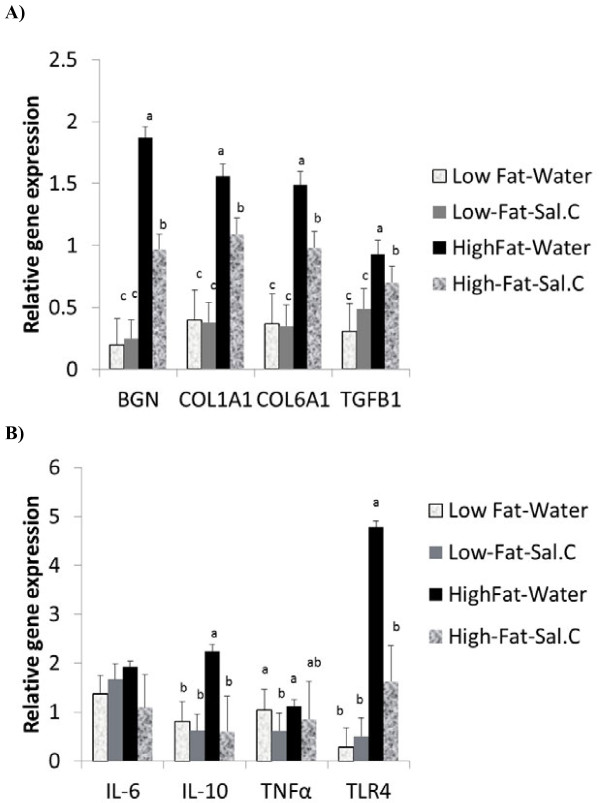
**Effect of sodium salicylate (Sal.C) treatment on expression of genes in mice on low fat or high fat diet in the subcutaneous adipose tissue**. Subcutaneous RNA was analyzed for the expression of ECM genes (**a**) or cytokines (**b**). Bars represent means and SE of treatments (n = 8). Bars with different superscripts are different (P < 0.05).

In the subcutaneous AT (Figure 3), HFD upregulated the expression of key ECM genes (BGN, COL1A1 and COL6A1 (P < 0.0001). High fat diet also induced the expression of TGFβ1 (P < 0.002), IL-10 (P < 0.05) and TLR4 (P < 0.0005). However, sodium salicylate counteracted the effect of HFD and led to a significantly lower expression of BGN (P < 0.04) and COL6A1 (P < 0.01). Additionally, salicylate treatment downregulated the expression of TNFα (P < 0.0006) and TLR4 (P < 0.07). Therefore, although HFD induced the expression of these genes, salicylate treatment effectively minimized HFD-induced subcutaneous AT expansion with an attendant downregulation of inflammatory and ECM gene expression.

**Figure 4:**
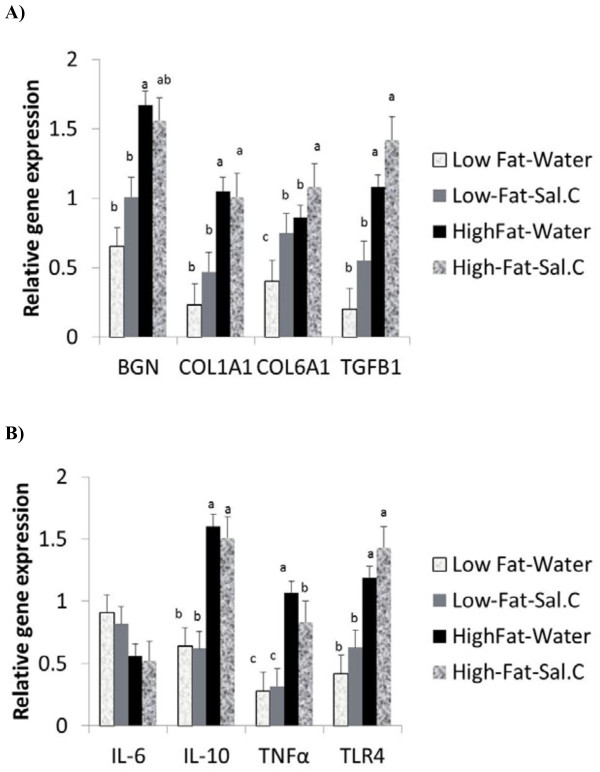
**Effect of sodium salicylate (Sal.C) treatment on expression of genes in mice on low fat or high fat diet in the epididymal adipose tissue**. Epididymal tissue RNA was analyzed for the expression of ECM genes (**a**) or cytokines (**b**). Bars represent means and SE of treatments (n = 8). Bars with different superscripts are different (P < 0.05).

**Figure 5:**
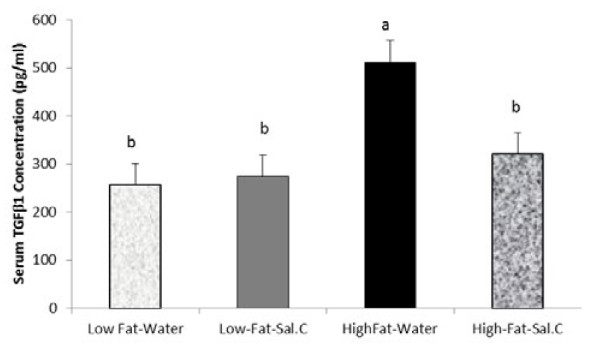
**Effect of diet and sodium salicylate (SS) treatment on serum TGFβ1 concentration**. Male C57BL/6J mice fed either a control (CON) diet (10% fat calories) or a high fat diet (HFD) (60% fat calories) for 8 weeks and provided with either plain water (Normal) or sodium salicylate (25mg/100ml). Fasting TGFβ1 level was determined with ELISA. Bars represent means and SE of treatments. Bars with different superscripts are different (P < 0.05).

In the epididymal AT (Figure 4), HFD induced the expression of BGN, COL1A1, COL6A1, TGFβ1, TNFα, IL-10, TLR4 (P < 0.0001) and IL-6 (P < 0.03). However, there was a reduced overall effect of sodium salicylate in the epididymal adipose depot compared to the subcutaneous depot. Nevertheless, salicylate reduced the circulating concentration of TGFβ1, a profibrotic protein involved in the induction of many ECM genes including biglycan, indicating that it could have an overall anti-fibrotic effect (Figure 5).

## Discussion

Our study has shown that the increased inflammation in obesity also involves elevated expression of ECM genes in AT. The strong association between biglycan expression and that of inflammatory genes supports a major role for this protein in AT inflammation. Indeed, as we have shown previously [9], reduced expression of TNFα was found in the mesenteric AT of biglycan knockout mice. We have extended this finding to include other inflammatory genes, IL-6 and CD68. In other experiments (report in preparation), we have shown that biglycan directly stimulate the expression of IL-6 in adipocytes, providing a confirmatory evidence that biglycan may indeed play a critical role in the expression of inflammatory genes in AT. Possible mechanism of biglycan action could include direct activation of TLR4 or the inflammasome pathways [5,8].

Although this work and our earlier report [9] clearly shows that obesity or HFD induces the expression of biglycan in AT, the possible implication of inflammatory state in the regulation of biglycan expression is unknown. We have shown that treatment with an anti-inflammatory agent sodium salicylate led to a reduced subcutaneous AT mass and inflammation that was associated with a concurrent downregulation of ECM genes. Salicylate based compounds inhibit the gene expression and protein levels of IL-6, IL-8 and TNFα in 3T3-L1 adipocytes [19]. In this study, salicylate treatment downregulated the expression of inflammatory cytokines (IL-6 and TNFα), perhaps due to its effect in reducing the expression of TLR4. A strong positive association between NF-kB and TLR4 expression is reported in monocytes of diabetic subjects [20] and the downregulation of expression of TLR4 could represent a new anti-inflammatory mechanism of effect of salicylate, in addition to its direct inhibition of NFkB. The downregulation of TLR4 by salicylate could have far reaching consequence for the prevention of inflammation in AT. For example, it is well established that free fatty acids and other pathogen derived ligands signal through the TLR4 in AT and perpetuate inflammation manifested in increased expression of inflammatory cytokines such as IL-6 and TNFα [21]. Therefore, downregulation of TLR4 by salicylate could contribute to the reduced expression of IL-6 and TNFα observed with the salicylate treatment. An elevated level of interleukin-10, an anti-inflammatory cytokine, is commonly found in obesity to counteract the effects of inflammatory cytokines [22]. The finding of lowered IL-10 by salicylate may suggest that, by lowering the inflammatory state, less of the anti-inflammatory action of IL-10 was needed in the presence of salicylate.

Although several studies have linked AT ECM gene expression with inflammatory state [6,23], none has explored a therapy aimed at reducing both events simultaneously. As we had hypothesized, salicylate downregulated the expression of BGN, COL6A1, and COL1A1and lowered inflammatory gene (IL-6, TNFα and TLR4) expression in the subcutaneous AT. This confirms the coupling of inflammation to ECM gene expression and the importance of targeting inflammatory events in AT for obesity and fibrosis prevention. The relationship between ECM gene expression and inflammation appears complex. Inflammatory environment upregulates the expression of several ECM genes in human preadipocytes [24]. On the other hand, there is evidence that ECM genes such as biglycan and fibronectin are associated with induction of inflammation [8,25,26]. Therefore, lowering inflammation with salicylate prevents the induction of ECM gene expression by inflammatory factors. The downregulation of ECM genes will ultimately lead to reduced adipose mass as was observed in the subcutaneous AT depot. In this study, we did not observe a pronounced effect of salicylate on the epididymal AT mass and gene expression. The more robust effect of HFD and the dampened effect of salicylate on ECM and cytokine gene expression in the epididymal depot reflect a potential difference between the adipose depots and may also indicate either a need for higher dose of salicylate or longer duration of treatment to observe significant changes in the epididymal tissue. However, this also raises a possibility that the effect of anti-inflammatory compounds may first be observed in the subcutaneous AT rather than epididymal AT. It is well recognized that the subcutaneous adipose depot typically has reduced inflammatory gene expression than visceral depot [27-29]. This could be due to the reduced adipocyte size in this depot or may reflect a higher sensitivity to anti-inflammatory mechanisms. In conclusion, targeting inflammatory pathways in AT may be an effective strategy for suppressing ECM gene expression and for reducing AT mass and the inflammation associated with adipocyte expansion.

## Abbreviation

ECM = Extracellular matrix; ELISA = Enzyme linked immunoabsorbent assay; HFD = High fat diet; IL-6 = Interleukin 6; NFκB = Nuclear factor kappa B; TGFβ = Transforming growth factor β; TLR = Toll-like receptors; TNF = Tumor necrosis factor.

## Competing interests

The authors declare that they have no competing interests.

## Authors’ contributions

KMA designed and obtained primary funding for the study. VJA conducted the animal experimentation, laboratory and statistical analysis with help from MW. All authors participated in evaluation of study finding and development of the article. All read and approved the final manuscript.
